# Supplementary material for: Multi-view clustering via global-view graph learning
Source: PLoS One. 2025 Jun 2;20(6):e0321628. doi: 10.1371/journal.pone.0321628 (PMC12129154; doi:10.1371/journal.pone.0321628)
Supplement: Attachment — Submitted filename: Response to reviewers.docx [file pone.0321628.s001.docx]

[Manuscript ID]: PONE-D-24-45409

[Title]: Multi-view Clustering via Global-view Graph Learning

[Authors]: Dr. Qin Li and Geng Yang

[Date]: 2024.11.23

**Dear Editor,**

We would like to thank the academic editor and reviewers for their constructive feedback on our manuscript. Below, we provide detailed responses to all comments and explain how we have addressed them in the revised manuscript.

**Comments from Academic Editor:**

Comment 1: Include specific hardware details and execution times to substantiate efficiency claims.

Response: We have added detailed hardware specifications in the revised manuscript. Specifically, we performed all experiments on a CPU-only machine with the following specifications: Intel Xeon Platinum 8352V CPU (2 processors, 2.10GHz base frequency, 3.50GHz turbo frequency) and 128GB RAM. No GPU was used, further validating the efficiency of our proposed method. Execution times for all datasets have been provided in Table 1 to substantiate our efficiency claims.

Comment 2: Refine language and structure for readability.

Response: We have thoroughly proofread the manuscript to improve clarity and readability (see Section see Section ‘abstract’, ‘introduction’, ‘parameter analysis’, and ‘conclusion’). Additionally, we separated the discussion of performance metrics and theoretical insights into distinct paragraphs for better organization (see Section ‘discussion’).

Comment 3: Add visual aids (such as convergence curves) to illustrate performance.
Response: We have added convergence curves to visually demonstrate the performance of our proposed algorithm (see Fig. 1 in Section ‘Convergence Behavior’). These curves provide a clear representation of the algorithm's convergence behavior across different datasets, enhancing the interpretability of our results.

Comment 4: Briefly acknowledge any limitations, like parameter sensitivity.

Response: We have added a dedicated section in the discussion to acknowledge the limitations of our method, particularly its sensitivity to the parameterβ. This parameter balances the contributions of global and view-specific information, and its improper tuning can affect clustering performance. To address this limitation, we are going to explore automatic parameter selection or parameter-free approaches in future work to enhance the robustness of the algorithm.

**Comments from Reviewer #1:**

Comment 1: Provide more detailed hardware configurations, including CPU, RAM, and GPU specifications, along with execution times.

Response: We have added detailed hardware specifications in the revised manuscript. Specifically, we performed all experiments on a CPU-only machine with the following specifications: Intel Xeon Platinum 8352V CPU (2 processors, 2.10GHz base frequency, 3.50GHz turbo frequency) and 128GB RAM. No GPU was used, further validating the efficiency of our proposed method. Execution times for all datasets have been provided in Table 1 to substantiate our efficiency claims.

Comment 2: Refine sentences for greater clarity.

Response: We have thoroughly proofread the manuscript to improve clarity and readability (see Section ‘abstract’, ‘introduction’, ‘parameter analysis’, and ‘conclusion’).

Comment 3: In certain sections, such as the Discussion, the combination of different topics (e.g., performance and theoretical explanation) makes the text harder to follow. Separating these topics into distinct paragraphs would improve clarity.

Response: We separated the discussion of performance metrics and theoretical insights into distinct paragraphs for better organization (see Section ‘discussion’).

Comment 4: Alongside numerical performance metrics, incorporating visual elements such as convergence curves or clustering accuracy plots would make the results more intuitive and accessible to readers.

Response: We have added convergence curves to illustrate the optimization process and t-SNE-based clustering visualization plots for the Yale and ORL datasets to demonstrate the clustering performance visually. These visualizations provide an intuitive understanding of the algorithm's behavior and effectiveness. (see Fig. 1 in Section ‘Convergence Behavior’ and Fig. 2 in Section ‘Visualization of Clustering Results’).

**Comments from Reviewer #2:**

Comment 1: Some theoretical aspects of the method could benefit from further elaboration. For example, the process of constructing the Global Affinity Matrix and how it captures the "complementary information" from multiple views is only briefly mentioned. A more detailed breakdown of this step, with mathematical explanations, would help readers understand how the model works and why it performs well compared to others.

Response: We have elaborated on the process of constructing the Global Affinity Matrix and provided a detailed mathematical explanation of how it captures complementary information from multiple views. This additional clarification ensures a deeper understanding of the underlying mechanisms and the strengths of our method (see Section 'Problem Formulation and Objective Function').

Comment 2: The paper discusses adaptive learning of weight parameters for different views, but the explanation lacks clarity. Providing a more intuitive explanation, possibly with examples, on how the weights are dynamically adjusted for each view would improve comprehension.

Response: We have elaborated on the process of constructing the Global Affinity Matrix and included a detailed mathematical explanation of how it captures complementary information from multiple views. Additionally, we clarified how the weight parameters for different views are dynamically adjusted to ensure optimal integration of view-specific and global information. These improvements enhance the reader's understanding of the underlying mechanisms and the strengths of our method (see Section 'Problem Formulation and Objective Function').

Comment 3: The Related Work section could benefit from a clearer separation between different types of multi-view clustering approaches. Grouping similar methods (e.g., spectral clustering, subspace clustering) would make the review more coherent and provide a better context for the proposed method’s novelty.

Response: Upon reviewing our manuscript, we realize that the comment likely pertains to the Introduction section rather than the Related Work section, as the introduction contains a more detailed review of various multi-view clustering methods. To address this, we have restructured the Introduction section by grouping methods into distinct categories such as spectral clustering-based methods, subspace clustering-based methods, and other advanced approaches. This reorganization enhances clarity and provides a more coherent context for understanding the novelty of our proposed method. The updated structure now clearly highlights how our method builds on and addresses the limitations of these existing approaches. (See the revised Introduction section in the manuscript for details.)

Comment 3: The discussion on performance metrics and theoretical insights could be separated for better clarity. Mixing performance analysis with theoretical explanations makes it harder to follow. A more distinct division of topics would enhance readability.

Response: We separated the discussion of performance metrics and theoretical insights into distinct paragraphs for better organization (see Section ‘discussion’).

Comment 4: Including graphs that show the convergence behavior of the proposed method, as well as a comparison of runtime versus dataset size, would visually reinforce the efficiency claims. These plots would allow readers to see how quickly the model converges and how it scales with different dataset sizes.

Response: Thank you for the suggestion. We have included graphs demonstrating the convergence behavior of the proposed method and a comparison of runtime versus dataset size. These additions visually reinforce the efficiency claims and provide readers with a clearer understanding of how quickly the model converges and how it scales with varying dataset sizes. (See Section 'Convergence Behavior' and Table 1 in the revised manuscript for details.)

Comment 5: A brief discussion of the method's limitations, such as its sensitivity to certain parameters, would provide a more balanced view and offer directions for future improvements.

Response: We have added a dedicated section in the discussion to acknowledge the limitations of our method, particularly its sensitivity to the parameterβ. This parameter balances the contributions of global and view-specific information, and its improper tuning can affect clustering performance. To address this limitation, we are going to explore automatic parameter selection or parameter-free approaches in future work to enhance the robustness of the algorithm.

We hope that the revised manuscript addresses all comments and suggestions effectively. Thank you for your consideration.

Sincerely,

Qin Li and Geng Yang

The Shenzhen Institute of Information Technology
