# Supplementary material for: Multi-view clustering via global-view graph learning
Source: PLoS One. 2025 Jun 2;20(6):e0321628. doi: 10.1371/journal.pone.0321628 (PMC12129154; doi:10.1371/journal.pone.0321628)
Supplement: Attachment — Submitted filename: Response_to_reviewers_auresp_2.docx [file pone.0321628.s002.docx]

[Manuscript ID]: PONE-D-24-45409

[Title]: Multi-view Clustering via Global-view Graph Learning

[Authors]: Dr. Qin Li and Geng Yang

[Date]: 2024.11.23

**Dear Editor,**

We would like to thank the academic editor and reviewers for their constructive feedback on our manuscript. Below, we provide detailed responses to all comments and explain how we have addressed them in the revised manuscript.

**Comments from Academic Editor:**

Comment 1: Thank you for submitting your manuscript to PLOS ONE. After careful consideration, we feel that it has merit but does not fully meet PLOS ONE’s publication criteria as it currently stands. Therefore, we invite you to submit a revised version of the manuscript that addresses the points raised during the review process.

Response: Thank you for your consideration of our manuscript and for providing us with the opportunity to revise and resubmit. We appreciate the constructive feedback from the reviewers, which has helped us improve the clarity and rigor of our work. In response to the reviewers' comments, we have carefully revised our manuscript to address all the concerns raised.

**Comments from Reviewer #1:**

Comment 1: This paper proposes an efficient multiview spectral clustering method that constructs a Global-view Graph to integrate complementary information from different views. Unlike standard spectral clustering, their method directly assigns cluster labels without post-processing, such as K-means. After revisions, this paper meets the criteria for acceptance.

Response: Thank you for your positive feedback and for recognizing the contributions of our work. We sincerely appreciate your time and effort in reviewing our manuscript. Your constructive comments have been invaluable in improving the clarity and rigor of our research.

**Comments from Reviewer #3:**

Comment 1: On page 6, in Equation (11), how is (11) derived from (9)? The Frobenius norm of \( ||X||_F = \sqrt{\sum_{i,j} X_{ij}^2} \), not merely the sum of all elements.

Response: Thank you for your valuable comment. There was indeed a writing error in the derivation, leading to an inaccurate expression of the Frobenius norm. The norm should be defined based on the sum of squared elements rather than merely summing all elements. I have carefully reviewed and corrected the derivation of Equation (11) from Equation (9) to ensure its accuracy. I sincerely appreciate your thorough review and insightful feedback, which have greatly helped improve the clarity and correctness of the paper. (Please refer to Section 'Optimization').

Comment 2: On page 6, in Equation (12), are the weights \( W_v \) and \( W \) guaranteed to be nonnegative?

Response: Thank you for your insightful comment. Equation (12) is related to Equation (11), but due to a writing error in Equation (11), the formulation of Equation (12) was also affected. As a result, the nonnegativity of the weights was not guaranteed. I have carefully reviewed and corrected both equations to ensure their correctness. I sincerely appreciate your thorough review and valuable feedback.(Please refer to Section ''Optimization'').

Comment 3: In Equations (17) and (6), what is the difference between \( S_i^T \cdot 1_n = 1 \) and \( s_i 1_n = 1 \)?

Response: Thank you for your valuable feedback regarding the constraints in Equations (6), (7), (8), and (17). I acknowledge that there was an inconsistency in the notation used to represent the row-wise normalization condition due to an oversight in the original formulation. Following your suggestion, I have carefully revised these four equations to ensure consistency in their expression. I sincerely appreciate your insightful comments, which have helped improve the clarity and precision of the paper. (Please refer to the revised section “Parameter-Weighted Multi-View Clustering (PwMC)” and “Self-Weighted Multi-View Clustering (SwMC)” in the manuscript for details.)

Comment 4: On pages 7 and 8, does the proof establish Lemma 1? Additionally, how does it show the convergence of Algorithm 1?

Response: Thank you for your insightful comment. Based on your guidance, we have carefully revisited and rederived the entire proof to ensure that Lemma 1 is properly established and to clarify how it demonstrates the convergence of Algorithm 1. We sincerely appreciate your valuable feedback, which has helped us improve the rigor and clarity of our work. (Please refer to the revised section “Converge Analysis” in the manuscript for details.)

Dear Editor and Reviewers, we sincerely appreciate your valuable feedback, which has helped us improve the manuscript. We hope that our revisions effectively address all comments and suggestions. Thank you for your time and consideration.

Sincerely,

Qin Li and Geng Yang

The Shenzhen Institute of Information Technology
